# Occupational Health Needs and Predicted Well-Being in Office Workers Undergoing Web-Based Health Promotion Training: Cross-Sectional Study

**DOI:** 10.2196/14093

**Published:** 2020-05-26

**Authors:** Devan Richard Tchir, Michael Lorne Szafron

**Affiliations:** 1 School of Public Health University of Saskatchewan Saskatoon, SK Canada

**Keywords:** eHealth, health promotion, occupational health, well-being, internet, workplace

## Abstract

**Background:**

Office workers face workplace-related health issues, including stress and back pain, resulting in considerable cost to businesses and health care systems. Workplace health promotion attempts to prevent these health issues, and the internet can be used to deliver workplace health promotion interventions to office workers. Data were provided by Fitbase GmbH, a German company, which specializes in workplace health promotion via the internet (Web-based health). The Web-based health intervention allowed workers to focus on different health categories by using information modules (reading health information) and/or completing practical exercises (guided, interactive health tutorials).

**Objective:**

This study aimed to identify the extent to which office workers have workplace-related health issues, assess whether office workers who differ in their health focus also differ in their improved well-being, and assess whether completing practical exercises is associated with improved well-being compared with reading information modules.

**Methods:**

Fitbase GmbH collected data for the period of February 2016 to May 2017 from health insurance employees undergoing Web-based health training in Hamburg, Germany. The data consisted of a needs assessment examining health issues faced by office workers, a wellness questionnaire regarding one’s perception of the Web-based health intervention, and activity logs of information modules and practical exercises completed. Through logistic regression, we determined associations between improved well-being from Web-based health training and differences in a worker’s health focus and a worker’s preferred intervention method.

**Results:**

Nearly half of the office workers had chronic back pain (1532/3354) and felt tense or irritated (1680/3348). Over four-fifth (645/766) of the office workers indicated that the Web-based health training improved their well-being (*P*<.001). Office workers who preferred practical exercises compared with information modules had 2.22 times greater odds of reporting improved well-being from the Web-based health intervention (*P*=.01; 95% CI 1.20-4.11). Office workers with a focus on practical exercises for back health had higher odds of improved well-being compared with other health foci. Office workers focused on practical exercises for back pain had at least two times the odds of having their well-being improved from the Web-based health intervention compared with those focused on stress management (*P*<.001), mindfulness (*P*=.02), stress management/mindfulness (*P*=.005), and eye health (*P*=.003). No particular health focus was associated with improved well-being for the information modules.

**Conclusions:**

Office workers frequently report having back pain and stress. A focus on Web-based health training via practical exercises and practical exercises for back health predict an improvement in office workers’ reported well-being.

## Introduction

### Background

Office workers face a multitude of health issues, including back pain [1], stress [2,3], eye problems [4], and upper limb pain [1]. Internationally, costs from back pain [5] and stress in the workplace [6] are extensive. Eye problems decrease the productivity of office workers [7], and upper extremity injuries result in long absences from work and are a financial burden to businesses [8]. All of these health afflictions can have a significant financial toll through loss of work and increased health care expenses.

Workplace health promotion can be used to prevent many workplace-related health concerns and can include a range of information delivery systems. These systems include intervention programs, disease management programs, print materials, health education classes, fitness facilities on worksites, health fairs, and interventions for diet and fitness, among other methods [9]. Web-based electronic health interventions (Web-based health) are also available, which can be used to address the stated health issues. Web-based health interventions have been shown to decrease back pain [10], help manage stress [9,11], and provide improvements to nutritional and physical activity habits [9,12]. Addressing health issues via Web-based health techniques is favorable given that they can be personalized, highly structured, visually stimulating, and readily available to workers [13]. Furthermore, internet-based interventions have been shown to dramatically restore work capacity [14], have more favorable changes in health behavior and knowledge compared with non–Web-based interventions [15], and improve worker well-being [16].

Self-reported well-being and self-reported health have been beneficial predictors for decreased sick days, decreased costs to the health care system, and increased productivity [17-20]. There is also evidence that well-being is negatively associated with spinal pain [21], stress [22], eye problems [23], and mortality [24]. Thus, self-reported well-being as an outcome of Web-based health training can be an important measure. If a worker’s reported well-being differs from his or her health concern (such as stress management or back pain) is an important question. Although there is literature documenting the effects of Web-based health interventions on worker well-being (through measures of stress, psychological distress, depression scores, etc) [16], we found little literature documenting the effect of Web-based health interventions on a worker directly reporting that their well-being had been improved from an intervention (defined in this work as *self-reported well-being*). Furthermore, many Web-based health interventions to our knowledge aim to improve a worker’s psychological well-being through cognitive therapies and stress management [16], not via physical health improvement (ie, back health).

Web-based health promotion interventions primarily aim to change health behaviors through education and may involve the worker passively reading or listening to health information without engaging workers in practical, hands-on exercises designed to improve health. The effectiveness of practical, hands-on exercises (as opposed to simply reading information modules) in Web-based interventions is undocumented in the literature to the best of our knowledge. Also, information on self-perceived health issues (related to back pain, stress, etc) for office workers goes largely unreported.

### Objectives

Our study was designed to address these two gaps in the literature. This study has the following research objectives: (1) identify the extent to which office workers have health issues related to repetitive strain injury (RSI, ie, upper limb pain associated with computer work), back pain, resilience, mindfulness, nutrition, stress, and eye health; (2) to assess if office workers who differ in their health focus also differ in their self-reported well-being; and (3) evaluate if completing practical exercises influences the self-reported well-being of office workers more than reading information modules.

## Methods

### Data

Data regarding health insurance employees in Hamburg, Germany, undergoing Web-based health training for the period of February 2016 to May 2017, was sourced from Fitbase GmbH, a German company based out of Hamburg, Germany, specializing in Web-based health training for companies and individuals. Their Web-based health training intervention consists of reading educational information modules (with health categories in back pain, stress management, nutrition, mindfulness, eye health, RSI, and resilience), and practical exercises (with health categories in back pain, stress management, mindfulness, eye health, and RSI). The information modules educate workers on maintaining a healthy lifestyle, and the practical exercises are guided health tutorials for activities related to healthy behavior. The data included responses to a needs assessment, a questionnaire, and practical exercise and information module logs of the employees. For privacy reasons, no employee demographic information was made available.

The total number of employees enrolled in the Web-based health training was 5694. The needs assessment was administered before beginning the Web-based health training and consisted of statements associated with RSI, back pain, resilience, mindfulness, nutrition, stress management, and eye health. These statements were, respectively: (1) “After intensive PC (personal computer) work, I feel pain, tingling or numbness in my hands or arms”; (2) “I have upper and lower back pain”; (3) “I basically assume that I can overcome difficulties in life”; (4) “I notice that I’m lost in thought about the future or the past”; (5) “I crave fast food”; (6) “I feel tense or irritated”; and (7) “My eyes hurt after work.” Responses to these statements were *never*, *rarely*, *sometimes*, *frequently*, and *constantly*. Questionnaires regarding perceptions of the Web-based health training were administered during training at roughly 3, 6, and 9 weeks and were completed voluntarily. The last questionnaire completed was used for all analyses to best capture an individual’s perception at the end of training. The statement of interest on the questionnaire was “Online training improves my well-being,” answered by a worker as *strongly disagree*, *disagree*, *agree*, or *strongly agree*. The University of Saskatchewan Research Ethics Board waived the need for ethics approval based on secondary analyses of the data.

### Variables

#### Outcome Variable

Those who responded *strongly disagree* or *disagree* to the statement on the questionnaire “Online training improves my well-being” were coded as *disagree*, and those who responded *agree* or *strongly agree* were coded as *agree*, creating a binary outcome variable. These responses were combined due to a small sample size.

#### Independent Variables: Preferred Type of Intervention, Practical Exercise Health Focus, and Information Health Focus

The practical exercises completed and information modules read throughout the Web-based health training were recorded per user. On the basis of these logs, we computed the total numbers of practical exercises completed, and information modules read per health category. From these totals, we computed per individual the grand total of practical exercises completed and the grand total of the information modules read. We then defined a user’s *preferred type of intervention* as the intervention associated with the greater of these two grand totals; if these grand totals were not different, the workers *preferred type of intervention* was set to be *no preference*. For example, if a worker completed statistically more practical exercises than information modules, they were assigned a *practical exercises* preference.

A user’s *practical exercise health focus* was defined to be the practical exercise health category associated with the maximum total of the practical exercise-related health categories. For example, if a worker completed most of their practical exercises in stress management, they were assigned a *stress management* focus. A user’s *information health focus* was derived similarly. If two health categories were completed most frequently, a tie was assigned to the worker. The number of ties was negligible (each combination <1%) except for workers completing both stress and mindfulness, and both back pain and RSI practical exercises. Consequently, we respectively labeled their *practical exercise health focus* as *stress and mindfulness* and *back and RSI*, and we excluded the data for individuals with all other ties.

### Analyses

To address our first research objective (which was to identify the extent office workers experience health issues such as RSI, back pain, resilience, mindfulness, nutrition, stress, and eye health), we summarized the frequencies of the needs assessment data. From these frequencies we found the proportion of office workers who responded as *never*, *rarely*, *sometimes*, *frequently*, and *constantly* to the questions/statements: (1) “After intensive PC work, I feel pain, tingling or numbness in my hands or arms” (n=3354); (2) “I have upper and lower back pain” (n=3354); (3) “I basically assume that I can overcome difficulties in life” (n=3348); (4) “I notice that I’m lost in thought about the future or the past” (n=3354); (5) “I crave fast food” (n=3354); (6) “I feel tense or irritated” (n=3348); and (7) “My eyes hurt after work” (n=3348).

To address our second and third objectives, we developed a logistic regression model that predicted the well-being of workers from the Web-based health training. Those who completed at least two practical exercises, two information modules, and a questionnaire were included for the logistic regression analysis (n=779). The response to the statement, “Online training improves my well-being,” was used as the outcome variable. Independent variables included the derived variables *practical exercise health focus*, *information health focus*, and *preferred type of intervention*. Office workers with an *RSI* categorized *information health focus* (n=7) and with a *back and RSI* categorized *practical exercise health focus* (n=6) were removed due to the extremely small sample size and were not included in the final model (n=766). Due to insufficient sample size, interactions between variables were not considered in the model.

Bivariate analyses of the independent variables with the outcome variable having *P*<.20 were to be included in the model, and all subsequent analyses were completed at the alpha=.05 level. All variance inflation factor scores were below 1.7, indicating a negligible influence of multicollinearity. All statistical analyses were conducted using SAS version 9.4.

## Results

### User Statistics

Of the 934 workers who completed a questionnaire, 765 (81.9%) indicated that the Web-based health training improved their well-being whereas 169 (18.1%) indicated the Web-based health training had not improved their well-being (*P*<.001).

Of the workers who completed two or more practical exercises, 29.69% (1057/3560) focused on back pain, 21.94% (781/3560) focused on stress management, 18.93% (674/3560) focused on mindfulness, 13.88% (494/3560) focused on stress and mindfulness equally, 10.81% (385/3560) focused on eye health, and 2.70% (96/3560) focused on RSI (Table 1).

Of the workers who completed two or more information modules, 46.07% (2415/5242) focused on back pain, 18.41% (965/5242) focused on stress management, 17.89% (938/5242) focused on nutrition, 7.34% (385/5242) focused on mindfulness, 5.32% (279/5242) focused on eye health, 2.61% (137/5242) focused on RSI, and 2.35% (123/5242) focused on resilience (Table 2). Among the workers who completed a questionnaire and did two or more information modules and practical exercises, 84.2% (645/766) reported that the Web-based training had improved their well-being (*P*<.001; Table 3).

**Table 1:** Number of office workers categorized in each practical exercise health focus who completed at least two practical exercises (n=3560), and the number of office workers categorized in each practical exercise health focus who completed at least two practical exercises, at least two information modules, and a questionnaire (n=779).

Health focus	Office workers categorized in each practical exercise health focus who completed practical exercises (N=3560), n (%)	Office workers categorized in each practical exercise health focus who completed information modules, practical exercises, and a questionnaire (N=779), n (%)
Back pain	1057 (29.69)	226 (29.0)
Stress management	781 (21.94)	213 (27.3)
Mindfulness	674 (18.93)	162 (20.8)
Stress and mindfulness	494 (13.88)	76 (9.8)
Eye health	385 (10.81)	73 (9.4)
RSI^a^	96 (2.70)	23 (3.0)
Back and RSI	73 (2.05)	6 (0.8)

^a^RSI: repetitive strain injury.

**Table 2:** Number of office workers categorized in each information health focus who completed at least two information modules (n=5242), and the number of office workers categorized in each information health focus who completed at least two information modules, at least two practical exercises, and a questionnaire (n=779).

Health focus	Office workers categorized in each information health focus who completed information modules (N=5242), n (%)	Office workers categorized in each information health focus who completed information modules, practical exercises, and a questionnaire (N=779), n (%)
Back pain	2415 (46.07)	345 (44.3)
Stress management	965 (18.41)	234 (30.0)
Nutrition	938 (17.89)	96 (12.3)
Mindfulness	385 (7.34)	51 (6.6)
Eye health	279 (5.32)	24 (3.1)
RSI^a^	137 (2.61)	7 (0.9)
Resilience	123 (2.35)	22 (2.8)

^a^RSI: repetitive strain injury.

**Table 3:** Descriptive statistics of the variables considered for the logistic regression model and bivariate logistic regression analyses of the independent variables with the outcome variable “online training improves my well-being” (n=766).

Independent variable		Number of workers (N=766), n (%)	*P* value
**Practical exercise health focus**			.003
	Back pain	224 (29.2)	
	Stress management	211 (27.6)	
	Mindfulness	162 (21.2)	
	Stress and mindfulness	76 (9.9)	
	Eye health	72 (9.4)	
	RSI^a^	21 (2.7)	
**Information health focus**			.12
	Back pain	344 (44.9)	
	Stress management	233 (30.4)	
	Mindfulness	51 (6.7)	
	Nutrition	92 (12.0)	
	Eye health	24 (3.1)	
	Resilience	22 (2.9)	
**Preferred type of intervention**			.002
	Practical exercises	208 (27.2)	
	Information modules	351 (45.8)	
	No preference	207 (27.0)	
**Worker reports that online training improves their well-being**			N/A^b^
	Strongly agree/agree	645 (84.2)	^ ^
	Strongly disagree/disagree	121 (15.8)	

^a^RSI: repetitive strain injury.

^b^N/A: not applicable.

### Research Objective 1

Our first research objective was to identify the extent to which office workers have health issues related to different health categories. Our results related to this are the distributions of the responses to the needs assessment questions as illustrated in Table 4. Over 42.60% (1429/3354) never felt pain, tingling, or numbness in their hands or arms after intensive PC work. Nearly half of the office workers (1532/3354) chronically had back pain. About three-fourth (2554/3348) believed they can consistently overcome difficulties in life. Half of the office workers (1680/3348) felt chronically tense or irritated. About one-fifth of office workers (643/3348) had eye pain after work frequently or more often.

### Research Objectives 2 and 3

The results of the logistic regression model, including *P* values, odds ratios (ORs), and 95% CIs, could be found in Table 5. For our second research objective, which was to assess if office workers who differ in their health focus differ in their self-reported well-being, we found one’s *practical exercise health focus* to be statistically significant (Wald chi-square value, χ^2^_5_=14.5; *P*=.01) with a worker’s improved well-being from the Web-based health training. For our third research objective, which was to evaluate if completing practical exercises influence self-reported well-being more than information modules, we found one’s *preferred type of intervention* to also be statistically significant (Wald chi-square value, χ^2^_2_=6.7; *P*=.03) with a worker’s improved well-being from the Web-based health training.

**Table 4:** Needs assessment frequency data of office workers in Germany. Number (%) represents the office workers and their responses to needs assessment statements (n=3354).

Needs assessment statements	Never, n (%)	Rarely, n (%)	Sometimes, n (%)	Frequently, n (%)	Constantly, n (%)	Missing, n (%)
“I basically assume I can overcome difficulties in life.”	15 (0.45)	147 (4.38)	632 (18.84)	1655 (49.34)	899 (26.80)	6 (0.18)
“I notice that I'm lost in thought about the future or the past.”	392 (11.69)	936 (27.91)	1025 (30.56)	787 (23.46)	214 (6.38)	0 (0.00)
“After intensive PC work, I feel pain, tingling or numbness in my hands or arms.”	1429 (42.61)	880 (26.24)	671 (20.01)	325 (9.69)	49 (1.46)	0 (0.00)
“I have upper and lower back pain.”	145 (4.32)	618 (18.43)	1059 (31.57)	1080 (32.20)	452 (13.48)	0 (0.00)
“I crave fast food.”	180 (5.37)	768 (22.90)	1115 (33.24)	967 (28.83)	324 (9.66)	0 (0.00)
“I feel tense or irritated.”	54 (1.61)	399 (11.90)	1215 (36.23)	1302 (38.82)	378 (11.27)	6 (0.18)
“My eyes hurt after work.”	747 (22.27)	1003 (29.90)	955 (28.47)	538 (16.04)	105 (3.13)	6 (0.18)

**Table 5:** The associations of different health foci and interventions with the well-being of office workers in Germany undergoing Web-based health training (n=766).

Independent variables		OR^a^ (95% CI)	1/OR^b^	*P* value
**Practical exercise health focus**				.01^c^
	Back pain (reference category)	N/A^d^	N/A	N/A
	Stress management	0.322 (0.166-0.623)	3.109	<.001
	Mindfulness	0.439 (0.215-0.895)	2.278	.02
	Stress/mindfulness	0.319 (0.143-0.710)	3.137	.005
	Eye health	0.291 (0.131-0.648)	3.436	.003
	RSI^e^	0.548 (0.142-2.124)	1.824	.38
**Preferred type of intervention**				.03^c^
	Information modules (reference category)	N/A	N/A	N/A
	Practical exercises	2.218 (1.196-4.112)	0.451	.01
	No preference	1.083 (0.671-1.749)	0.923	.74
**Information health focus**				.37^c^
	Back pain (reference category)	N/A	N/A	N/A
	Stress management	1.097 (0.652-1.848)	0.911	.73
	Mindfulness	0.704 (0.295-1.684)	1.42	.43
	Nutrition	0.643 (0.354-1.168)	1.556	.15
	Eye health	1.574 (0.411-6.029)	0.635	.51
	Resilience	1.979 (0.419-9.359)	0.505	.39

^a^OR: odds ratio.

^b^1/OR: reciprocal of the odds ratio.

^c^*P* value estimated from a Type III Analysis of Effects via a Wald χ^2^ test.

^d^N/A: not applicable.

^e^RSI: repetitive strain injury.

Compared with workers focused on practical exercises for back pain, workers focused on eye health, mindfulness, stress management, and stress/mindfulness statistically differed (refer to Table 5 for the respective *P* values). Those workers with a focus on practical exercises for back pain had 3.11 times the odds greater than those focused on stress management (*P*<.001), 2.28 times the odds greater than those focused on mindfulness (*P*=.02), 3.14 times the odds greater than those focused on stress/mindfulness (*P*=.005), and 3.44 times the odds greater than those focused in eye health practical exercises (*P*=.003) of having their well-being improved.

Among office workers whose preferred intervention was practical exercises, the odds were 2.22 times greater to report improved well-being from the Web-based health training than those who preferred information modules (*P*=.01).

## Discussion

### Principal Findings

This study sought to examine the level of importance in which office workers perceived health issues, and examined possible differences in improved well-being from Web-based health training. We were interested in if there are differences in the associations for a worker’s well-being based on specific health categories and if completing guided practical exercises or reading educational information modules had a different impact on improving an office worker’s well-being.

In our exploration of office worker work-related health issues, back pain and stress management were most important; mindfulness and nutrition were moderately important; and RSI, resiliency, and eye health were of least concern to office workers. Back pain and stressful demands from work are common among office workers [25]. This aligns with the popularity of these two health categories in our study, as they were the most popular practical exercises and information modules completed by workers. Studies have reported a wide range of back pain prevalence (ranging from 23% to 56%) in office workers [1,26-29]. This is consistent with our finding that workers frequently focused on back pain when completing both practical exercises and information modules (29.69% and 46.07%, respectively).

Office workers may suffer from stress [2,3], and our study cohort was no different. Half of the office workers in this study felt chronically tense or irritated. However, over three-quarters of office workers in our study stated that they frequently or constantly could overcome life difficulties, suggesting our study participants are intrinsically confident in their abilities to overcome stressors. This indicates an apparent dichotomy between a worker experiencing stress and their resilience.

Oha et al [27] found that 7% of computer workers reported frequent wrist/hand or shoulder pain. Other studies report varying results; the prevalence of upper limb symptoms as low as 10% and as high as 52% have been documented in office workers [1,27-30]. Our findings support that workers experience varying degrees of pain after PC work, whereas most studies simply report if workers experience upper limb pain in a dichotomous fashion [1,28-31]. In fact, those responding *constantly feeling pain after PC work* might have disabling pain or worse [32]. In our study, 11.15% of workers chronically felt pain in their hands or arms after PC work, compared with 68.84% of workers reporting rarely or never. These estimates are considerably different from the literature, which estimates a higher prevalence of RSI pain [29]. Only 19.21% of our office workers reported routinely feeling eye pain after work. Another study identifying health issues in data processing office workers report a 26% prevalence of sore eyes from work [28].

Our logistic regression model yielded that well-being was influenced by different health categories. Workers focused on practical exercises for back pain had higher odds of having their well-being improved compared with almost all health categories. Prior literature has demonstrated that Web-based back pain interventions for office workers improves their quality of life [10], possibly consistent with our findings. Our logistic regression model also yielded that well-being was influenced by if office workers completed more practical exercises or more information modules. In general, completing practical exercises improved a worker’s odds of having their well-being improved. Our findings suggest that completing practical exercises can be more effective in improving well-being as opposed to passively reading information modules, thus suggesting a need for hands-on, instructional, do-it-yourself exercises in Web-based health promotion for the office workplace.

Furthermore, participation rates in Web-based health promotion activities are typically low and hence a major concern [33]. Evidence suggests that guided Web-based health programs promote worker retention [16]. Our finding that office workers have increased odds of improved well-being when utilizing guided, follow-along exercises might reflect this. Available literature shows exercise-driven back pain interventions can decrease pain and improve back function [34,35]. Our study suggests back health might be in greatest demand by office workers; this makes sense, given that many different occupations are linked with back pain [36]. For groups with limited resources, their worker’s health might benefit the most from focusing on back health.

Overall, an emphasis on practical exercises for back pain might benefit a workplace to the greatest extent when focusing on a worker population’s well-being. For our office worker population, we found that back pain was the most popular health focus and was perceived as a persistent health issue (although those who focused on practical exercises for back pain had higher odds of having their well-being improved). It is possible that different working populations have different health needs. Our work may suggest that workers should address their primary health concern with practical exercises for maximal improvement in their perceived well-being.

To the best of our knowledge, our findings are the first demonstrating that completing Web-based health training is associated with improved self-reported well-being in office workers and that completing practical exercises rather than simply receiving Web-based health information may improve well-being. Our work supports that practical exercises for Web-based health interventions are beneficial to Web-based health promotion as a whole, regardless of a specific health focus, in office workers. With respect to Web-based health promotion in the workplace, our work suggests that actively engaging workers to take part in their health is more beneficial than passively engaging workers. The data suggests that Web-based health interventions for an office-workplace should tailor resources toward promoting back health and in general, exercise-intensive health promotion strategies.

Given the potential cost benefits of reported well-being, employers and health insurers could benefit from incorporating practical back health exercises into Web-based health promotion efforts. As there is a shift in employment to many sedentary and office-confined occupations [37] and the internet is widely used globally [38], our findings might be applicable to a wider spectrum of occupations.

### Limitations

As only those who completed a questionnaire, practical exercises, and information modules were included in our logistic regression modeling, there is potential selection bias. The absence of demographic information on the office workers prevented us from controlling for socio-demographic confounding factors. The findings were centered on German office workers and therefore may not generalize to other countries. Health categories, from which workers chose, were not exhaustive; there are likely other important concerns that were not included in this study, such as physical activity [39]. Future studies should identify objective measures (blood pressure, cortisol levels, etc) and a way to compare the perceived quality of the practical exercises to information modules.

### Conclusions

Our finding suggests that a Web-based health promotion intervention using hands-on activities can be an effective method for addressing workplace-related health concerns and general worker well-being. We recommend future Web-based health promotion workplace interventions should focus on the main health issues experienced by workers and to include guided practical exercises for those who are wanting to improve their health. As technology becomes more mainstream in the workforce and jobs continue to shift toward seated computer work, Web-based health promotion will become increasingly more important.

## Abbreviations

OR: odds ratio
PC: personal computer
RSI: repetitive strain injury
